# Prevalence and correlates of disability in Bogra district of Bangladesh using the rapid assessment of disability survey

**DOI:** 10.1186/s12889-015-2202-7

**Published:** 2015-09-07

**Authors:** Manjula Marella, Nafisa L. Huq, Alexandra Devine, Sally M. Baker, Md A. Quaiyum, Jill E. Keeffe

**Affiliations:** Nossal Institute for Global Health, The University of Melbourne, Melbourne, Australia; Centre for Reproductive Health, International Centre for Diarrheal Disease Research, Bangladesh (icddr,b), Bangladesh; LV Prasad Eye Institute, Hyderabad, India

## Abstract

**Background:**

The aim of this study was to estimate the prevalence of disability and its associated risk factors among adults aged 18 years and over in Bogra district, Bangladesh.

**Methods:**

The Rapid Assessment of Disability (RAD) survey was conducted using probability-proportional-to-size sampling to select 66 clusters each with 50 people aged 18 years and older in 2010. Households within clusters were selected through compact segment sampling. Disability was identified based on the responses to the self-assessment of functioning section of the RAD questionnaire. Descriptive and multivariate logistic regression analyses were performed to model the associations between risk factors and disability status.

**Results:**

Of 1855 adults who participated in the study, 195 (10.5 %) had disability. Age and gender adjusted prevalence of disability in Bogra district was 8.9 % (95 % CI: 7.7, 10.3). The highest prevalence of functional limitation was related to psychological distress (4.7 %; 95 % CI: 3.8, 5.7) followed by vision (4.4 %; 95 % CI: 3.6, 5.4), and hearing (2.3 %; 95 % CI: 1.7, 3.0) difficulties. The adjusted odds of disability increased with age with approximately eight-fold increase from 2.9 % (95 % CI: 1.6, 5.1) in 18–24 years to 24.5 % (95 % CI: 20.2, 29.4) in 55 years and above. People with poor socio-economic status (OR 1.90; 95 % CI: 1.1, 3.3) and who were unemployed (OR = 4.6; 95 % CI: 1.8, 11.6) were more like to have disability compared to the higher socio-economic status and those who have an occupation respectively.

**Conclusions:**

There is a significant need for promoting programs for health, well-being, and rehabilitation, and policies specifically targeting the older population, women, unemployed and poor people in Bangladesh.

## Background

With growing evidence on the link between poverty, disability and equity, disability is recognized as a significant development issue [1]. Following the adoption of the United Nations Convention on the Rights of Persons with Disabilities (UNCRPD) [2] several governments, non-government organizations and international donors are increasingly committed to ensuring the development sector is inclusive of people with disability [3]. Reliable data on disability prevalence and associated risk factors are needed in order to plan disability inclusive development strategies, programs and policies [4, 5]. The Washington Group on Disability Statistics (WG) developed a set of questions to measure disability in adults based on the International Classification of Functioning, Disability and Health (ICF) framework [6] that conceptualizes disability as an outcome of the interaction between the health condition and contextual factors [7]. The WG questions cover the most basic actions or functions such as vision, hearing, walking, remembering, communication and self-care to measure disability in censuses and surveys.

Bangladesh is a low income country with a population of approximately 150 million, who live predominantly in rural areas [8]. Similar to other Asian countries, Bangladesh is experiencing a demographic transition with decreasing fertility rates and increasing life expectancy rates resulting in a growing older population [9]. Based on data from Matlab, a rural area of Bangladesh, mortality rates due to non-communicable diseases were shown to have significantly increased from 8 % in 1986 to 68 % in 2006 [10]. As a consequence of this ageing population and increasing prevalence of chronic conditions such as cardiovascular disorders and diabetes, the number of people with disability in Bangladesh is likely to increase [11].

There are different estimates of disability prevalence reported in Bangladesh, arising from different methodologies. This lack of reliable data makes it challenging to plan disability inclusive development strategies and policies. The Bangladesh national census surveys in 1982, 1986, 1998 and 2010 estimated the prevalence of disability to be 0.6, 0.5, 1.6 [12] and 1.4 % [8] respectively. Titumur and Hossain, reported a disability prevalence of 5.6 % based on a population based survey conducted in six divisions of Bangladesh in 2004 [12]. The census surveys and the survey by Titumur and Hossain considered all ages and used direct questions regarding speech, hearing, vision and intellectual impairments that considers disability as a consequence of a health condition that limits a person’s activities (Table 1). Mitra and Sambamoorthi [13] and the World report on disability [4] estimated a disability prevalence of 22.0 and 31.9 % respectively in Bangladesh based on different estimation methods using data from the World Health Survey (WHS) 2002–2004. While an ICF-based approach was used in the WHS, hearing and communication impairments were not considered. Another limitation of the WHS was that the estimated prevalence could be an overestimate, as respondents were asked about difficulties experienced during the last 30 days prior to the interview, which may have meant responses included short-term conditions [13]. The Household Income and Expenditure Survey (HIES) conducted in Bangladesh in 2010 used the WG short set questions and reported a disability prevalence of 9.1 % [14]. While the WG short set questions were designed for adults, the HIES used them for children as well. None of the earlier studies in Bangladesh have considered psychological distress in their measures of disability (Table 1).

**Table 1:** Disability surveys in Bangladesh

Authors	Study	Sample	Ages	Disability measures	Prevalence
Bangladesh Bureau of Statistics [8]	Census 2010	National census	All ages	Direct questioning on speech, vision, hearing, physical, mental and autistic disabilities.	1.4 %
Titumur and Hossain [12]	Disability in Bangladesh: Prevalence, Knowledge, attitude and Practices, 2004.	13,025 individuals sampled throughout the country.	All ages	Direct questioning on hearing, speech, vision, physical, and intellectual impairments.	5.6 %
Mitra and Sambamoorti [13]	World Health Survey 2002–2004	5,931 households and 5,549 individuals sampled throughout the country.	18 years and above	4 questions: seeing, moving around, concentrating or remembering, and self-care.	22.0 %
World report on Disability [4]	World Health Survey 2002–2004	5,931 households and 5,549 individuals sampled throughout the country.	18 years and above	16 questions on vision, cognition, affect, interpersonal relationships, mobility, pain, sleep and energy and self-care.	31.9 %
Bangladesh Bureau of Statistics [14]	Household Income and Expenditure Survey 2010	12,240 households sampled throughout the country.	All ages	WG short set (seeing, hearing, walking and climbing, remembering or concentrating, self-care, and communication)	9.1 %
Cherry et al. [20]	Gonoshasthaya Kendra survey 2010	43417 individuals from 600 villages.	60 years and above	12 questions based on WG questions: seeing, hearing, mobility, cognition, self-care, and communication domains.	26.0 %
Marella et al. (current study)	Rapid Assessment of Disability Survey 2010	2315 individuals in Bogra district.	18 years and above	15 questions based on WG questions: vision, hearing, mobility, communication, gross and fine motor, cognition, appearance and psychological distress.	8.9 %

The differences in disability prevalence are due to the different age ranges, methodologies and the areas where the surveys were implemented

Reliable data are needed on the prevalence of disability in Bangladesh in order to plan realistic and effective disability inclusive development strategies. More information is also needed on the factors associated with disability in Bangladesh to advocate and plan for policies and programs, such as social protection programs, which would positively impact on associated factors and ultimately improve the quality of life of people with disability. The aim of this study was to investigate the prevalence of disability and the socio-economic factors associated with disability among people aged 18 years and older using data from field testing of the Rapid Assessment of Disability (RAD) survey conducted in the Bogra District of Bangladesh [15]. The RAD survey collects data on the prevalence of disability based on activity limitations similar to the WG questions and also measures the impact of disability on a person’s well-being and access to the community [15].

## Methods

### Study design and sampling

A cross-sectional population-based survey using two-stage cluster random sampling was conducted in the Bogra district in 2010. The Bogra district of Rajshahi division is in northern Bangladesh. The 2010 Census estimated the population to be 3,000,000 [8]. There are 12 Upazilas (sub districts), with the agriculture and livestock sectors contributing to the majority of the economy. The literacy rate of Bogra district is low at 49.4 % [16], with 6.7 and 16.6 % households living in the lower poverty (extremely poor) and upper poverty (moderately poor) lines respectively [17].

A sample size of 3,300 people (all age groups) was estimated using the most recent estimate of disability prevalence at the time of this study, i.e. a disability prevalence of 5.6 % in the population [12], a 95 % confidence level, sampling error of 20 %, an estimated design effect of 1.75, and a non-response rate of 10 %. This sample required 66 clusters with 50 people in each cluster. Only findings from the adult sample (i.e. people aged 18 years and above) are presented in this paper. Of the 2315 eligible adult participants identified for the survey, 1855 (80 % response rate) were interviewed from 66 clusters. Among the non-respondents, 458 were away due to work or travelling outside the cluster location; only two refused to participate.

The sampling frame comprised villages (in rural areas) and mahallas (in urban areas) in Bogra, using population data from the 2001 national census projected to 2010. In the first stage, clusters (villages or mahallas) were selected through probability proportion to size sampling. The second stage involved selecting households within clusters through compact segment sampling. Each village and mahalla was divided into equal segments through mapping of the sites so that each segment comprised approximately 50 people. Segments to be included in the study were selected by randomly drawing lots. All households in the segment were included in the sample sequentially until 50 people were recruited. If fewer than 50 participants were recruited in a given segment, sampling continued in the next nearest segment until 50 people were recruited in a cluster. At least two return visits were made to absentees. In the absence of a head of the household, the next head of the household responded to the household questionnaire.

### Questionnaire

The RAD questionnaire [3] is interviewer administered and has two parts: a household questionnaire administered to the head of the household and an individual questionnaire administered to each individual in the household. The household questionnaire is comprised of questions related to household socio-economic characteristics such as source of water, having electricity, sanitation facility, roof, wall and floor materials; ownership of durable goods (e.g. television, radio, bicycle and motorcycle); and ownership of the house, land and cattle.

The individual questionnaire is comprised of four sections 1) demographics, 2) self-assessment of functioning, 3) well-being, and 4) access to the community [3, 15].

The demographics section includes items related to age, gender, education, and occupation. The self-assessment of functioning section is comprised of 15 items related to functioning in eight domains: vision (one item), hearing (one item), mobility (one item), communication (one item), gross and fine motor (one item), cognitive (three items), interacting with others due to appearance (one item) and psychological distress (six items) [3]. The nine items related to sensory/mobility/cognitive domains (functional limitation) were adapted from the Washington Group questions for measuring disability [7]. The psychological distress domain is an adaptation of the Kessler-6 scale, a short scale of mental health designed to assess the level of distress in clinical and population surveys [3, 18].

Each item in the self-assessment of functioning section asks participants to report the frequency of difficulty in functioning in the last 6 months even when using assistive devices available to them (e.g. seeing even if wearing glasses). The response categories are ‘none,’ ‘some of the time,’ ‘most of the time,’ and ‘all of the time.’ Disability was considered to be present in respondents who reported difficulty ‘most’ or ‘all of the time’ to at least one item related to the nine items related to functioning, and/or distress reported on at least two out of six items from the psychological distress domain. The rationale for using this cut-off criteria has been described in an earlier publication [15].

This paper presents the socio-economic factors associated with disability among adults, using data from the household questionnaire, and the demographics and self-assessment of functioning sections of the individual questionnaire. Further details on the development and testing of other sections of the RAD questionnaire, and findings from the well-being and access to the community sections have been reported in earlier publications [3, 15].

### Statistical analysis

Statistical analyses were performed using PASW Statistics 18 (PASW Statistics for Windows, SPSS Inc., Chicago, IL). Chi square and Fisher’s exact tests (used when any expected frequency was less than 5) were performed to determine whether disability is associated with demographic and socio-economic characteristics. Odd ratios were used to describe the strength of association between independent variables age of respondent, gender, education level, occupation and asset quintiles and the binary outcome variable, i.e. presence or absence of disability.

Multivariate logistic regression models were performed to assess the presence of statistically significant associations between socio-demographic risk factors and prevalence of disability by calculating odds ratios and 95 % confidence intervals (CI).

Age was grouped into five categories (18–24, 25–34, 35–44, 45–54 and 55 and over), completed years of education into four categories (none, 1–4 years, 5–9 years and 10 years or more) and occupation into five categories (unemployed, farmer, daily wage laborer, housewife and professional/others). Asset index was used as a proxy indicator for wealth status using principal components analysis on the data from the household questionnaire [19]. Individuals were ranked according to the asset index of the household in which they resided. The households were then divided into quintiles, with the first quintile representing the poorest in the sample, and the fifth quintile representing the wealthiest. The reference groups were 18–24 years, female, fifth quintile, more than 10 years of education and unemployed. Confidence intervals (CI) for prevalence estimates and regression odds ratios were calculated with adjustment for clustering effects in the study design using the generalized estimating equation approach. Age and gender adjusted prevalence rates were derived using projected population estimates for 2010 as the reference standard.

### Ethics approval

Ethics approval was obtained from the University of Melbourne Human Research Ethics Committee (Australia), and the International Centre for Diarrheal Disease Research, Bangladesh (icddr,b) Ethical Review Committee (Bangladesh). The study was conducted in accordance with the tenets of the Declaration of Helsinki. All participants provided written or verbal informed consent and did not receive any incentive for participation. When participants who were not literate their verbal consent was obtained, i.e. the consent form was read to participants and their verbal agreement was recorded by the interviewer in front of a witness. This protocol was approved by ethics committees.

## Results

### Socio-economic characteristics

Table 2 summarizes the socioeconomic characteristics of the sample and shows the comparison between people with and without disability. The mean (±SD) age of the participants was 38.6 (±16.2) years. The majority of the participants were female (58.9 %). Education level was low with 36.6 % having never attended school and only 11.6 % with 10 or more years of schooling. Just over half the participants were housewives (54.1 %), and about one-third of participants were either farmers (19.3 %) or daily wage laborers (13.0 %). People with disability were more likely to be in the older age group, illiterate, unemployed and belong to the poorest quintiles compared to those without disability (all *p* < 0.001) (Table 2).

**Table 2:** Socio-economic characteristics and disability status

	Sample (*n* = 1855)	Disability (*n* = 195)	No disability (*n* = 1660)	p value
	n (%)	n (%)	n (%)	
Sex				
Male	763 (41.1)	83 (42.6)	680 (41.0)	
Female	1092 (58.9)	112 (57.4)	980 (59.0)	0.667
Age (years)				
18–24	406 (21.9)	11 (5.6)	395 (23.8)	
25–34	461 (24.9)	23 (11.8)	438 (26.4)	
35–44	379 (20.4)	40 (20.5)	339 (20.4)	
45–54	275 (14.8)	37 (19.0)	238 (14.3)	
≥55	334 (18.0)	84 (43.1)	250 (15.1)	<0.001
Education				
None	679 (36.6)	99 (50.8)	580 (34.9)	
1–4 years	372 (20.1)	42 (21.5)	330 (19.9)	
5–9 years	588 (31.7)	40 (20.5)	548 (33.0)	
10 years or more	216 (11.6)	14 (7.2)	202 (12.2)	<0.001
Occupation				
None	130 (7.3)	51 (26.6)	79 (5.0)	
Farmer	343 (19.3)	26 (13.5)	317 (19.9)	
Daily wage laborer	231 (13.0)	22 (11.5)	209 (13.2)	
Housewife	964 (54.1)	84 (43.8)	880 (55.4)	
Professional/others	113 (6.3)	9 (4.7)	104 (6.5)	<0.001
Religion				
Islam	1661 (89.5)	179 (91.8)	1482 (89.3)	
Hindu	194 (10.5)	16 (8.2)	178 (10.7)	0.277
Socio-economic status				
Poorest quintile	312 (16.8)	50 (25.6)	262 (15.8)	
Second quintile	326 (17.6)	43 (22.1)	283 (17.1)	
Third quintile	381 (20.5)	39 (20.0)	342 (20.6)	
Fourth quintile	398 (21.5)	28 (14.4)	370 (22.3)	
Richest quintile	437 (23.6)	35 (17.9)	402 (24.2)	<0.001

### Prevalence of disability

**Fig. 1:**
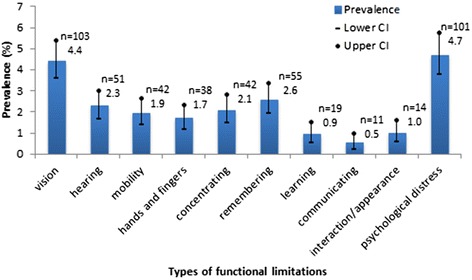
Prevalence of different types of functional limitations

A total of 195 people were identified to have disability, which is 10.5 % (95 % CI: 8.8, 12.2) prevalence of disability in the sample. The prevalence after adjusting for age and gender in the population was 8.9 % (95 % CI: 7.7, 10.3). The highest prevalence of functional limitation was related to psychological distress (4.7 %; 95 % CI: 3.8, 5.7) followed by vision (4.4 %; 95 % CI: 3.6, 5.4), and hearing (2.3 %; 95 % CI: 1.7, 3.0) difficulties (Fig. 1). Of the 195 people identified with disability, 99 (50.7 %) reported psychological distress in addition to other types of functional limitations (physical/sensory/cognitive/communication).

### Socio-economic factors associated with disability

Table 3 shows the prevalence of disability and its associations with gender, age, socio-economic status, education level and occupation. The prevalence of disability was slightly higher in females (9.4 %; 95 % CI: 7.8, 11.2) than in males (8.5 %; 95 % CI: 6.8, 10.5), however, the difference was not statistically significant (*p* = 0.495). The prevalence significantly increased with age from 2.9 % (95 % CI: 1.6, 5.1) in 18–24 year age group to 24.5 % (95 % CI: 20.2, 29.4) in 55 years and above (Table 3). The prevalence was significantly higher among lower education level groups, unemployed and those in poorest quintiles.

**Table 3:** Association of risk factors and prevalence of disability among people aged 18 years and over in Bogra district, Bangladesh

	Sample (*N* = 1855)		
	Prevalence^a^ (95 % CI)	Unadjusted OR (95 % CI)	Adjusted OR (95 % CI)^b^
Overall	8.91 (7.73, 10.25)		
Sex			
Female	9.35 (7.79, 11.17)	1	1
Male	8.46 (6.77, 10.53)	0.90 (0.66, 1.23)	1.01 (0.48, 2.16)
Age (years)			
18–24	2.85 (1.57, 5.11)	1	1
25–34	4.64 (3.07, 6.97)	1.66 (0.79, 3.50)	1.90 (0.82, 9.71)
35–44	9.15 (6.64, 12.47)	3.44 (1.71, 6.92)	4.21 (1.82, 9.72)
45–54	13.16 (9.59, 17.79)	5.17 (2.56, 10.46)	6.36 (2.78, 14.55)
≥55	24.49 (20.15, 29.43)	11.07 (5.73, 21.36)	8.25 (3.49, 19.49)
Education			
10 years or more	4.72 (2.77, 7.91)	1	1
5–9 years	6.20 (4.51, 8.46)	1.34 (0.70, 2.55)	1.46 (0.67, 3.22)
1–4 years	9.34 (6.84, 12.64)	2.08 (1.09, 3.98)	1.51 (0.67, 3.40)
None	13.12 (10.80, 15.85)	3.05 (1.69, 5.53)	1.36 (0.59, 3.09)
Occupation			
Professional/others	6.62 (3.42, 12.41)	1	1
Housewife	7.84 (6.35, 9.65)	1.19 (0.58, 2.48)	1.01 (0.36, 2.83)
Daily wage laborer	8.02 (5.18, 12.21)	1.23 (0.53, 2.84)	0.84 (0.34, 2.10)
Farmer	5.73 (3.82, 8.50)	0.86 (0.38, 1.93)	0.57 (0.24, 1.33)
None	37.65 (29.46, 46.62)	8.75 (3.99, 19.20)	4.58 (1.80, 11.62)
Socio-economic status			
Richest quintile	7.23 (5.16, 10.03)	1	1
Fourth quintile	5.68 (3.89, 8.22)	0.77 (0.45, 1.32)	0.89 (0.50, 1.59)
Third quintile	8.85 (6.39, 12.12)	1.25 (0.75, 2.06)	1.47 (0.83, 2.60)
Second quintile	11.29 (8.37, 15.07)	1.63 (1.00, 2.67)	1.68 (0.94, 3.00)
Poorest quintile	13.30 (10.08, 17.35)	1.97 (1.22, 3.17)	1.90 (1.09, 3.30)

*OR* Odds ratios, *CI* Confidence intervals; All measures are adjusted for clustering. Values in bold represent independent variables found to be statistically (*p* < 0.05) associated with disability. ^a^Adjusted for age and gender. ^b^Adjusted for sex, age, socio-economic status, education and occupation

A multivariate logistic regression analysis to investigate correlates of disability prevalence incorporating age, gender, education, occupation and socio-economic status was found to be statistically significant against a constant model (chi square 147.0, *p* < 0.001 with df 14). The Wald criteria demonstrated that after adjusting for age, gender, education, occupation and socio-economic status the prevalence of disability was significantly associated (*p* < 0.05) with older age groups (from 35 years and above), the poorest quintile, and unemployment (Table 3). Prevalence of disability increased with age by approximately eight-fold increase in the 55 years and older age group compared to 18–24 years. The odds of having disability increased close to two-fold for those in the poorest quintiles compared to those in the richest quintile and increased four and half times among those who were unemployed compared to those in the professional/other employment category. No significant association was found between the prevalence of disability and gender and education level.

There was no evidence of interactions between age, occupation and socio-economic status, which could be due to small sample size. Further multiple regression analysis was conducted to investigate the associations between disability and socioeconomic factors among people aged 55 years and above (*n* = 334). After adjusting for gender, education, occupation and socio-economic status, disability in people aged 55 years and above was significantly associated with unemployment (OR 3.5; 95 % CI: 2.1, 6.1) and poor socioeconomic status (OR 2.1; 95 % CI: 1.2, 3.7).

## Discussion

The findings in this study are comparable with other studies in Bangladesh that have used similar types of functioning questions. The HIES conducted in 2010 estimated disability prevalence of 9.1 % using the WG short set questionnaire [14]. The most commonly reported functional limitation in the HIES were vision (5.6 %) and hearing (1.9 %). The disability prevalence found among people aged 55 years and above by RAD (25 %) was similar to the findings reported by Cherry et al., where 26 % of their sample of people aged 60 years and above from rural villages were found to have disability (Table 1) [20]. This similarity in estimates is probably due to the majority (90 %) of the sample in the RAD survey being from rural areas of Bogra district and similar type of functioning questions used in both surveys to determine disability. These findings indicate that even though same set of questions were not used, WG type of questions on functioning (specific to activity limitations) provide reliable data on disability prevalence.

Disability prevalence found using RAD was much lower than was reported using the WHS data in Bangladesh. Mitra and Sambamoorthi [13] estimated 22 % of disability prevalence in Bangladesh based on four questions of Washington Group questions (seeing, moving, remembering and self-care) (Table 1). As mentioned earlier, disability prevalence may have been overestimated in the WHS because respondents were questioned about difficulties in functioning experienced in the last 30 days, while the RAD asked people to identify difficulties experienced in the last 6 months. Compared to the WHS, the RAD survey might be more likely to pick up difficulties in function resulting from longer term health problems.

One of the strengths of the RAD survey is that it includes measurement of psychological distress related to anxiety and depression. Psychosocial disability is often not considered in disability surveys despite growing recognition that people with psychosocial disability are a marginalized group who are often excluded from the disability movement and mainstream policies and programs. People with psychosocial disability experience significantly more discrimination and barriers in meeting their needs and priorities than people with other types of impairment [4]. Although not all components of psychosocial disability were included, this survey identified that nearly half of those who reported functional limitations also experienced psychological distress. This finding supports the growing recognition that people with psychosocial disability must be included in rehabilitation and development programs [21] and that rights of people with psychosocial disability should be supported by policies. It also emphasizes the need for promoting counselling, and other culturally accepted and rights based approaches to promoting positive well-being not only for people with psychosocial disability but also for other types of disabilities.

Tareque et al. reported disability was associated with age, sex, education, marital status, and place of residence using HIES data [22]. It was found that the disability prevalence was significantly higher in females (10.8 %) compared to males (8.8 %) in HIES. Although similar estimates were found in the current study, the difference between males and females was not statistically significant probably because of a smaller sample size. While the RAD survey had a good response rate, the majority of non-responders were away due to work or travelling. It may be possible that the non-responders were more likely to be male and non-disabled because they were participating in work outside home and were able to travel. Disability was more common among younger women compared to men (data not shown) and this may be due to consequences of inadequate health care [22]. Traditionally, women in Bangladesh are valued less than men in the community [23], and they usually have lower education and employment rates [24], and experience more chronic diseases compared to men [25]. Women are also at risk of health issues and impairments related to inadequate reproductive health care. The demographic trends show that women tend to live longer than men in Bangladesh [26]. In line with the findings from this study on the associations between disability and older age, unemployment and poverty, there is a need for strengthening existing policies and schemes that allocate a quota for women promoting their increased access to education, employment and health care.

One of the limitations of the current study is that data related to causes of functional limitations was not collected. Information on health conditions would have provided understanding of possible links between health conditions and disability, particularly in older age groups. Bangladesh is experiencing demographic transition with an increasing aged population, often associated with an increasing prevalence of chronic diseases [10]. Changing trends in family dynamics in Bangladesh also indicate elderly people will be more likely to be living in social isolation and poverty [25]. Data from this study show that people in older age groups with disability in Bogra district were more likely to be unemployed and live in poverty. The majority of medical and rehabilitation services/facilities in Bangladesh are concentrated in urban areas while the older population is more likely to be living in rural areas. There is a significant need for planning health, rehabilitation, social welfare and disability services for people with disability, particularly for the older population in rural Bangladesh. Community-based services targeting empowerment of the elderly and training of primary health care providers in managing disability in rural areas could address this need.

Having a disability was statistically significantly associated with an increase in the likelihood of being in the poorest two quintiles and being unemployed in this sample. These results are similar to other studies from Bangladesh that showed people with disability were more likely to be unemployed [27], and more likely to be poor [3, 21, 22]. Being a cross-sectional study design this survey is unable to demonstrate whether disability is a cause of unemployment and poverty. The relationship between disability and poverty has been described as cyclic in the literature highlighting people with disability are more likely to be poor, and higher rates of disability are associated with higher rates of poverty, unemployment and illiteracy [28–30]. Given this evidence from the literature, addressing barriers to employment for people with disabilities is an important consideration for disability inclusive development programs in Bangladesh. Although there are social protection schemes currently available for people with disability, a knowledge, attitudes and practices survey identified that the majority of people with disability were unaware of the benefits they were entitled to [31]. Therefore, there is a need for advocacy strategies for creating awareness on disability issues and rights of people with disability to social protection.

This study found that the level of education was poor among people with disability and it was statistically significantly associated with having disability on unadjusted regression model. However, a poor level of education was not found to be significantly associated with disability when adjusted for other factors (age, gender, occupation and socio-economic status). This result contradicts findings from HIES that showed educated people had significantly lower rates of disability compared to those who are uneducated even after adjusting for other socio-economic factors. The current study also contradicts Filmer’s findings from household survey data from developing countries where much of the association between disability and poverty was found to be mediated by education [29]. The difference is possibly because the HIES data included children as well and Filmer’s data included only adults aged 20–50 years, whereas the RAD survey included individuals aged 50 years and above who were more likely to be uneducated. The current study did not collect information on the onset of disability which could have provided information on how the age of onset of disability impacts on various life events such as access to education.

## Conclusions

In summary, this study provides reliable data on disability prevalence in Bogra district and shows older age, unemployment and poverty are significant risk factors for disability. This is the first study that has considered psychological distress as a contributory disability measure in Bangladesh and identified the need to include people with psychosocial disability in both disability specific (e.g. rehabilitation) and mainstream programs. Findings suggest that there is a significant need for strengthening programs for health, well-being and rehabilitation, and policies which create national mandates for the implementation of programs specifically targeting elderly, poor, women and unemployed people in Bangladesh.

## Abbreviations

CI: Confidence interval
HIES: Household Income and Expenditure Survey
ICF: International Classification of Functioning, Disability and Health
RAD: Rapid Assessment of Disability
SD: Standard deviation
UNCRPD: United Nations Convention on the Rights of Persons with Disabilities
WG: Washington Group on Disability Statistics
WHS: World Health Survey
